# Challenges and Progress for Treatment of Malignant Peripheral Nerve Sheath Tumors in the Context of Recent Successes for Sarcoma Therapy

**DOI:** 10.3390/cancers17233781

**Published:** 2025-11-26

**Authors:** John F. Callaghan, Raymond R. Mattingly

**Affiliations:** 1 Department of Pharmacology & Toxicology, Brody School of Medicine, East Carolina University, Greenville, NC 27834, USA; 2 Department of Pharmacology, School of Medicine, Wayne State University, Detroit, MI 48201, USA

**Keywords:** sarcoma, MPNST, neurofibromatosis, NF1, targeted therapy, immunotherapy, review

## Abstract

Malignant peripheral nerve sheath tumors (MPNSTs) are one of the most difficult sarcomas to treat. Due to the rarity of MPNSTs, many of the therapeutic approaches used are from treatment guidelines for soft tissue sarcoma. Besides surgery, little success has been achieved using these therapies. Traditional chemotherapy and radiation therapy regimens designed to treat sarcoma have unclear efficacy when used to treat MPNSTs. Targeted therapeutics that succeeded in other sarcomas failed to produce positive results in MPNSTs. Moreover, investigational agents that have shown efficacy in preclinical models have produced disappointing outcomes in clinical trials. While therapeutic options for patients with MPNST have remained relatively stagnant, dramatic improvements in therapeutic outcomes of other rare sarcomas have been made. This difference in success is likely caused by the complex heterogeneity of MPNSTs that hinders drug development, although many MPNSTs are associated with neurofibromatosis type 1 (NF1), a genetic disorder resulting from mutations in the NF1 gene that encodes the negative RAS regulator neurofibromin. The development of new agents for MPNST treatment has shifted away from solely targeting RAS pathway gene products to stimulating the immune system and manipulating other MPNST driver mutations such as *CDKN2A/B*, *SUZ12*, *EED*, and *TP53*. This review presents recent advances in the treatment of sarcomas and the future of drug development targeting MPNSTs.

## Simple Summary

Malignant peripheral nerve sheath tumors (MPNSTs) are rare soft tissue sarcomas commonly associated with genetic cancer predisposition neurofibromatosis type 1. These tumors are resistant to conventional chemotherapy and radiotherapy, leaving surgery as the only definitive treatment. Many drugs have been developed and investigated to improve the outlook for patients who develop MPNSTs, but outcomes have yet to significantly improve. During this same period, multiple other rare sarcomas have had innovative therapies developed that have greatly enhanced patient outcomes. These successes suggest a need for MPNST research to focus on additional driver mutations such as *CDKN2A/B*, *SUZ12*, *EED*, and *TP53* or immunotherapeutic approaches. Advancements in the treatment of other sarcomas can be used to help guide therapeutic development for MPNSTs, provide an understanding of approaches that are likely to succeed, and give hope for current and future patients.

## 1. Introduction

Malignant peripheral nerve sheath tumors (MPNSTs) are an aggressive form of cancer categorized as a soft tissue sarcoma (STS) by the World Health Organization [1]. MPNSTs are commonly defined as malignant tumors often arising from preexisting benign neurofibromas and ultimately from cells of the peripheral nerve sheath. They are responsible for 5–10% of all STSs diagnosed in the United States [2]. The generally accepted incidence of MPNSTs in the general population is approximately 1 in 100,000 [3]. However, the incidence has been reported to be as low as 1.46 in 1,000,000 for the adult population and 0.56 in 1,000,000 for the pediatric population, with incidence seeming to increase with age in each subgroup [4]. Approximately 50% of all MPNST cases are associated with neurofibromatosis type 1 (NF1), with the rest either sporadic or linked to irradiation [3].

NF1 is an autosomal dominant cancer predisposition caused by mutations in the *NF1* gene resulting in the loss of the tumor suppressor neurofibromin, which is a negative Ras regulator. NF1 has a birth incidence between 1 in 2000 and 1 in 3000, and patients with NF1 carry a lifetime risk of developing a MPNST between 8–13% [5,6,7]. The prognosis of patients with MPNST is poor, with the five-year survival rate reported to be between 21% and 69% [7,8,9,10,11]. The only observed predictors of patient outcomes are the location of the tumor and the size of the lesion at diagnosis [8,9]. According to some reports, patients with NF1 have worse clinical outcomes than patients with sporadic MPNSTs [8,12,13]. However, other studies suggest that the difference observed is not due to NF1 itself but instead other poor prognosis factors, such as larger tumor size at the time of diagnosis, which is commonly more exaggerated in patients with NF1 [11,14]. Whether NF1 is a poor prognosis factor for MPNSTs remains a topic of debate, and some recent studies suggest the gap in outcomes with sporadic tumors seems to be closing, though not all studies agree [11,12,15]. The most recent meta-analysis of MPNST survival outcomes after surgery found the overall survival (OS) to be 47% for MPNSTs and 50% for MPNSTs in the NF1 cohort [16]. However, when comparing survival for NF1-associated MPNSTs to sporadic cases from the same study, NF1-associated MPNSTs had an increased hazard ratio, which suggests that NF1 is a poor prognosis factor [16]. Regardless of whether NF1 status is a poor prognosis factor for MPNSTs or whether NF1-associated MPNSTs have had improved outcomes as time has passed, their treatment is difficult and overall patient outcomes remain poor.

MPNSTs are known to be resistant to both traditional chemotherapy and radiation therapy, leaving surgery as the primary form of treatment [17]. After surgery, MPNSTs have a recurrence rate between 30 and 70% [16,18,19,20]. Compared with MPNSTs, other STSs have an average recurrence rate of 35% and an average five-year survival rate of 60% [21,22]. In recent years, some sarcomas have had their treatment revolutionized by new therapeutics, while little success has been found so far in the treatment of MPNSTs.

## 2. Sarcoma Treatment

Sarcomas are a large group of rare heterogenous malignancies arising from mesenchymal cells. As a group, sarcomas have an incidence of between 4.2 and 7.7 per 100,000, with higher incidence rates being reported in the United States than in Europe [23,24,25,26,27]. However, they are responsible for less than 1% of all adult cancers [28]. Of these tumors, 85% are categorized as STSs and 15% as bone sarcomas [27]. Both groups have multiple histological subtypes. The most common subtypes of bone sarcoma are osteosarcoma (27.0–45.2%), chondrosarcoma (27.7–40.4%), and Ewing sarcoma (15.2–21.6%), while the most common STS are liposarcoma and leiomyosarcoma, each being responsible for approximately 20% of cases [27,29,30]. MPNSTs account for about 5–10% of all STSs, making them relatively common amongst the approximately 70 subtypes of STS [1,2,31]. Unfortunately, MPNST is also associated with one of the lowest disease-specific survival rates following surgical resection of primary STS, being below 50% [32].

### 2.1. Surgery

There are three main modalities of treatment for cancer, i.e., surgery, radiation, and systemic therapies. In early disease stages, cancer is typically treated with surgery alone. In later stages of the disease, treatment of cancer is often multi-modal and different combinations may be employed [33]. Surgery is the golden standard for localized disease, with radiotherapy and/or systemic therapy being used as neoadjuvant or adjuvant therapy. Neoadjuvant therapy is used preoperatively to shrink the mass of the tumor for more complete and safer resections, as well as perioperatively and postoperatively to lower the risk of recurrence [17]. In advanced disease states such as metastasis, surgery becomes less effective and systemic therapies can become the primary treatment [33]. In the case of localized sarcoma, surgery is a cornerstone of treatment, with the goal of a complete oncologic resection (R0). Alternatively, a microscopically positive resection (R1) is performed if an R0 resection is impossible, along with adjuvant and/or neoadjuvant radiotherapy as the first-line treatment [21,32,34]. In cases where limb sparing surgery is not possible, amputation is performed.

### 2.2. Radiation Therapy

Radiation is the second most successful form of curative cancer therapy behind surgery and is currently being used to treat approximately 50% of patients [35,36]. Curative radiotherapy can be used as an adjuvant, a neoadjuvant, or as the definitive treatment, while it is also used for palliative care. The goal of radiotherapy is to damage the DNA of the tumor cells by delivering a lethal dose of radiation. Radiation is used in the treatment of both bone sarcoma and STS. Sarcomas are often radioresistant, though radiosensitivity varies, resulting in radiotherapy only being used for high-risk and palliative cases [37,38]. The toxic effects of radiation are thought to have preferential lethality against malignant tissues compared with healthy tissues [35]. However, damage to DNA still occurs in healthy tissue and can result in the generation of a secondary cancer. Less than 10% of all secondary cancers are associated with radiotherapy and the risk of radiation-induced malignancy is below 1% for modern regimens [36,39]. Radiation-induced sarcomas (RISs) are responsible for 2.5–5.0% of all sarcomas, while radiation is responsible for approximately 10% of all MPNSTs, which comprises 5% of all RISs [40,41,42,43]. This is potentially due to patients with NF1 having a higher risk of tumor development due to their somatic haploinsuffiency in the *NF1* gene.

### 2.3. Chemotherapy

The primary systemic therapies are cytotoxic chemotherapy, targeted therapy, and immunotherapy. Cytotoxic chemotherapeutics primarily disrupt the cell cycle or damage DNA to prevent the proliferation needed to facilitate malignant growth. These agents are most often used in combination with drugs that have different mechanisms of action and spectrums of toxicity, allowing for synergistic activity and improved therapeutic outcomes [33]. Generally, chemotherapy is not considered curative and is used in the adjuvant or neoadjuvant setting in combination with surgery [33]. A recent review found that, for the treatment of STS, the anti-tumor antibiotic doxorubicin followed by the alkylating agent ifosfamide are the most efficacious and commonly used drugs [44]. They are often used in combination clinically and have been shown to increase the objective response rate (ORR) and the median progression free survival (PFS) of patients compared with either agent individually [44]. However, doxorubicin alone is still recommended as the first line agent for STS, as the combination therapy does not improve the median OS and increases certain hematologic toxicities [45,46,47]. Combination therapy is only recommended as a neoadjuvant to shrink tumors for curative surgery in high-risk STS [46,48]. Currently, no combination therapy has been shown to increase the median OS beyond doxorubicin alone for STS as a group [44]. Some other agents have been shown to have high efficacy against specific subtypes of STS such as the vinca alkaloids, which have substantial effects in rhabdomyosarcoma [44]. For the treatment of bone sarcoma, first line chemotherapy regimens combine cisplatin, doxorubicin, ifosfamide, and methotrexate, with surgery remaining the definitive treatment [49].

### 2.4. Targeted Therapies

Targeted therapies are drugs that specifically target molecules and signaling pathways that cancer cells rely on for survival and proliferation. However, the definition is sometimes broadened to include angiogenesis inhibitors and immunotherapeutic agents [50,51,52]. Targeted therapies are typically aimed at pathways that are mutated and/or upregulated and have genetic markers that can be used to predict if a specific tumor would be responsive to a specific therapy. Biomarkers found in tumors can then be used to personalize treatment to a specific patient or tumor type based on predicted responses [51]. Targeted therapies are similar to cytotoxic chemotherapeutics in that they are used as neoadjuvant, adjuvant, and palliative agents. Many recent research efforts in oncology have focused on the development of targeted therapeutics with reduced toxicity burden when compared with classical cytotoxic chemotherapeutics [51].

There are three main groups of protein kinases, being tyrosine kinases, serine/threonine kinases, and dual specificity kinases. Currently, the largest class of targeted therapeutics are tyrosine kinase inhibitors (TKIs), with over 50 approved by the FDA [53]. Since 2013 more than 10 new targeted agents have been approved by the FDA to treat various sarcoma or sarcoma-related tumors (Table 1). Seven of these new therapeutics are protein kinase inhibitors (PKIs). Of the seven protein kinase inhibitors recently approved for sarcoma, the majority are TKIs.

The first of these TKIs to be approved was regorafenib, which was accepted for the treatment of gastrointestinal stromal tumors (GISTs) in 2013 as a third-line agent behind imatinib and sunitinib. Imatinib is a small molecule TKI targeting BCR-ABL, KIT proto-oncogene, receptor tyrosine kinase (KIT), and platelet-derived growth receptor alpha (PDGFRA). Approximately 90% of GISTs have driver mutations in *KIT* and *PDGFRA*. In total, 85% of patients with advanced GISTs have been found to benefit from imatinib therapy [34,54]. Imatinib resulted in a median PFS of almost two years [54]. In cases where the disease is resistant to imatinib or there is patient intolerance, second-, third-, and fourth-line multi-TKI therapeutics have been approved. In a randomized phase III clinical trial, sunitinib was found to increase median patient PFS by 21 weeks compared with the placebo and could partially overcome imatinib resistance [55]. Regorafenib was shown to improve median PFS in patients with metastatic and/or unresectable GISTs after failure of imatinib and sunitinib [56]. Ripretinib has been approved for patients with continued progression or intolerance to the other agents. Median PFS was further extended 6 months, and the OS seemed to increase; however, the ORR compared with the placebo was negligible [57]. Avapritinib was also approved for GIST with PDGFRA D842V mutation, which was resistant to other agents [58].

In 2022 the FDA approved the use of crizotinib, an anaplastic lymphoma kinase (ALK) inhibitor, for inflammatory myofibroblastic tumors (IMTs), an ultra-rare sarcoma. Approximately 50% of all IMTs have gene rearrangements or fusions of the *ALK* gene, resulting in its overexpression and oncogenicity [59]. Clinical trials showed the ORR of crizotinib in adults to be ~70%, while it was ~86% in the pediatric population [60,61].

Three additional PKIs were also approved for sarcoma-related tumors. The CSF-1R inhibitor pexidartinib was approved for tenosynovial giant cell tumor (TGCT), while the MEK inhibitors selumetinib and mirdametinib were approved for inoperable symptomatic plexiform neurofibromas. TGCT is a rare tumor affecting joints and tendon sheaths that is normally benign, with its malignant variant either arising from a precursor tumor or de novo [62]. The CSF-1R inhibitor pexidartinib underwent clinical trials for only the benign variant of this tumor and was found to cause a robust regression of the tumor [63,64].

Plexiform neurofibromas are benign tumors associated with NF1 and are precursors to MPNSTs. Plexiform neurofibromas are thought to be congenital tumors in 30–50% of patients with NF1 [65,66,67,68]. Selumetinib and mirdametinib are distinct from the other recently approved PKIs as they inhibit mitogen-activated protein kinase kinase (MEK), which is a dual specificity protein kinase. Patients in pediatric clinical trials of selumetinib for symptomatic inoperable plexiform neurofibromas had durable tumor shrinkage and improvement in pain over a five-year treatment period, with 70% of patients having at least a partial response [69,70]. Mirdametinib had an ORR of 41% in adults and 52% in the pediatric cohort for treatment of plexiform neurofibromas. It was approved in 2025 for use in adults and pediatric patients, compared with selumetinib, which was approved in 2020 for the pediatric population and for the adult population in late 2025.

Nirogacestat is a γ-secretase inhibitor that was approved to treat desmoid tumors in 2023. Desmoid tumors are soft tissue tumors that are locally aggressive, with no metastatic potential. These agents were repurposed from Alzheimer’s disease to treat cancer due to their ability to inhibit the cleavage of Notch family proteins and blocking the Notch signaling pathway [71,72,73]. Desmoid tumors have activated Notch pathway signaling, which contributes to their oncogenicity [74]. Using nirogacestat to treat desmoid tumors resulted in an ORR of 41% compared with 8% in the placebo group [72]. This is a substantial improvement considering it is the first targeted therapy approved for desmoid tumors [72].

After the first clinical trial for malignant perivascular epithelioid cell tumor (PEComa), the mammalian target of rapamycin (mTOR) inhibitor nanoparticle albumin-bound sirolimus (*nab*-sirolimus) was approved for use by the FDA. *nab*-Sirolimus is an intravenous formulation of the mTOR inhibitor sirolimus [75]. PEComa is known to be related to genetic mutations of tuberous sclerosis complex (TSC) genes *TSC1* and *TSC2* [76]. TSC is a negative regulator of the mTOR pathway, thus mutations in TSC increase mTOR pathway signaling in PEComa [77,78]. The use of *nab*-sirolimus had an ORR of only 39%; however, 89% of patients with a *TSC2* mutation responded to the therapy. Overall, *nab*-sirolimus had a median PFS of 10.6 months and median OS of 40.8 months [75]. The survival seemed to increase compared with reported rates for general STS clinical trials [75].

The enhancer of zeste homolog 2 (EZH2) inhibitor, tazemetostat, was FDA-approved for the treatment of epithelioid sarcoma in 2020. EZH2 is an enzymatic catalytic subunit of the polycomb repressor complex 2 (PRC2), which is responsible for the epigenetic regulation of transcription through histone 3 trimethylation on Lys-27 (H3K27me3). EZH2 has been shown to methylate non-histone targets, such as STAT3, and to affect downstream genes, such as androgen receptor gene transcription independent of its role in PRC2 [79,80,81]. A subset of epithelioid sarcoma is characterized by a loss of *INI1/SMARCB1*, a component of the SWI/SNF complex that functions as a tumor suppressor antagonistic to PRC2 and is thought to allow for the oncogenic activation of EZH2 [82,83,84]. Treatment of epithelioid sarcoma with tazemetostat resulted in a median PFS of 5.5 months and a median OS of 19 months, both similar or improved compared with conventional chemotherapy [84].

In 2020, the FDA approved the use of pomalidomide for the treatment of Kaposi sarcoma. Kaposi sarcoma is a tumor that is often multicentric in nature and thought to be primarily caused by infection with Kaposi sarcoma-associated herpesvirus (KSHV) [85]. Pomalidomide exerts its antiangiogenic effects through inhibition of the VEGFR pathway, while its immunomodulatory effects stem from a costimulatory action increasing the population of CD4+ and CD8+ T-cells as well as natural killer cells [86,87,88]. Given that Kaposi sarcoma is characterized by abnormal neovasculature driven by upregulated VEGF signaling and that immunosuppression increases risk of development, both mechanisms of action have potential to be therapeutic [89]. Clinical trials showed that median PFS was 16.6 with an ORR of 73% and efficacy was seen in both HIV-positive and HIV-negative patients [90]. These trials also concluded that the success of pomalidomide therapy was dependent on both its antiangiogenic and immunomodulatory effects [90].

Olaratumab is a monoclonal antibody for PDGFRA that was approved by the FDA in 2016 for STS following promising early phase clinical trials suggesting improvements in median OS nearing one year [91]. A follow-up phase III confirmatory trial was negative, however, and it was withdrawn from the market in 2019 [92]. Further clinical trials have tested olaratumab in different combination therapies, with most results failing to show significant improvement in the patient population, though it has shown some promise in combination with pembrolizumab [93,94]. Current clinical trials are assessing its role as a potential radiotherapeutic (NCT06537596).

### 2.5. Immunotherapy

Progress in using immunotherapy as a modality of cancer treatment is developing rapidly and aims to use interactions between the immune system and tumor cells to stimulate the destruction and removal of cancerous cells. The immune system needs to be able to differentiate self from foreign antigens and does so through a tightly regulated balance of activation and inhibition of immune responses, leaving the body in a constant state of immune surveillance [95]. The evasion of this immune surveillance is one of the main hallmarks of cancer [96]. Tumors can be split into “cold” tumors that lack immune infiltration and “hot” tumors that are characterized by immune infiltration [95]. In cold tumors, antigen recognition and T-cell infiltration is prevented through mechanisms including reduced tumor immunogenicity, decreased dendritic cell maturation, suppressed T-cell activity, and blockade of T-cell migration [95]. In contrast, “hot” tumors contain cytotoxic T-cells and natural killer cells that can express inhibitory immune checkpoint molecules and may inhibit the activity of immunosuppressive cells in the tumor microenvironment (TME) [97]. Exploiting these immune responses with selective immunotherapeutic agents has revolutionized the treatment of many cancers.

Immune checkpoint inhibitors (ICIs) are the predominant immunotherapeutic strategy and primarily show efficacy against “hot” tumors. For an immune response to occur after T-cell antigen recognition, co-stimulatory signals from the antigen-presenting cell are needed. Co-inhibitory receptors also exist, predominately PD1 and CTLA4, which inhibit T-cell receptor signaling and, by extension, an immune response [98]. ICIs block co-inhibitory receptors, allowing for the disinhibition of immune responses. These drugs primarily have efficacy in tumors where an immune response already exists that does not create a new response [99]. Similar to targeted therapies, immunotherapies use biomarkers to predict if a tumor would be responsive to the therapy, such as the increased expression of programmed death-1 (PD-1) and its ligand (PD-L1) being predictive of therapeutic response to their respective inhibitors [97].

ICIs have been evaluated in sarcomas with varying degrees of success. Sarcomas are highly heterogenous in their TME, resulting in different responses depending on the type of sarcoma as well as the immunotherapeutic approach used [34]. Studies in sarcoma have predominantly focused on the use of PD-1 and PD-L1 inhibitors as over half of all sarcomas express these proteins, with nearly 100% of chordoma and epithelioid hemangioendothelioma expressing both, while barely 20% of synovial sarcoma and Ewing sarcoma expressed either [100]. Studies using these inhibitors showed vast differences in response based on the tumor. A study using the PD-1 inhibitor pembrolizumab showed 40% of patients with undifferentiated pleomorphic sarcoma responded, while only 20% of patients with liposarcoma, 10% of patients with synovial sarcoma, and none of the patients with leiomyosarcoma responded [101]. While in bone sarcoma it was found that 20% of patients with chondrosarcoma, 5% of patients with osteosarcoma, and none of the patients with Ewing’s sarcoma responded [101]. Atezolizumab, a PD-L1 inhibitor, was FDA-approved in 2022 for the treatment of alveolar soft part sarcoma (ASPS). ASPS was found to respond to ICIs and have an ORR of over 50% in combination with a TKI; however, the size of the study was small [102,103]. Atezolizumab has an ORR of 37% and a PFS of 20.8 months, with few high-grade adverse effects [104].

The use of other forms of immunotherapy for treatment of sarcoma are still under investigation, with adoptive cell therapy (ACT) recently showing a breakthrough. In ACT, T cells are isolated from the patient’s tumor and then expanded in vitro, selecting for cells that can overcome the tumor. In August 2024, Afamitresgene autoleucel, a MAGE-A4 antigen T-cell receptor-based ACT, was approved for use in the treatment of specific unresectable or metastatic synovial sarcoma with an ORR of 43.2% in clinical trials, and an ORR ranging from 44 to 61% seen in preclinical studies [105,106,107]. Other ACTs such as chimeric antigen receptor T-cell therapy and tumor-infiltrating lymphocyte therapy or cancer vaccines have either yet to be extensively tested in STS or have so far been unsuccessful [108].

## 3. MPNST Therapy

MPNSTs are considered one of the more difficult sarcomas to treat and currently have limited therapeutic options. As an STS, many of their treatment standards are taken from other STS. Thus, the goal of surgery is an R0 resection with adjuvant/neoadjuvant radiation or cytotoxic chemotherapy regimens centered on ifosfamide and/or anthracyclines, such as doxorubicin, sometimes with etoposide [109,110,111,112,113,114]. In cases where R0 resection is impossible in the extremities, limb-sparing procedures followed by irradiation are preferred; however, amputation offers superior survival in such cases [110,115,116]. In cases where an R0 resection is impossible for tumors in the head, neck, or trunk regions of the body, surgery is performed with the goal of an R0 resection followed by adjuvant therapy [110]. R0 resection remains the only curative treatment for localized MPNST; however, the risk of recurrence is high [15,117,118,119].

Cytotoxic chemotherapy and radiation therapy are both considered controversial for the treatment of MPNSTs. A retrospective study of MPNSTs between 1989 and 2017 in the Netherlands was unable to show an association between radiation therapy or cytotoxic chemotherapy and survival outcome [120]. The current chemotherapy regimens are taken from STS standards, and a retrospective analysis of European Organisation for Research and Treatment of Cancer trials found response rates for MPNST to be similar to other sarcomas in adult patients, with an ORR of 21% and a median PFS of 17 weeks [121]. It has been shown that ifosfamide + doxorubicin increased survival probability at one year from the start of treatment compared with other first-line treatments used, including etoposide-based therapies [17]. The European Paediatric Soft Tissue Sarcoma Study Group found that, among the pediatric population, the ORR was 46.2% and that NF1 was a poor prognosis factor for survival but did not affect the response rate [122]. The SARC006 clinical trial observed similar results for NF1 status, with 44.4% of sporadic MPNSTs responding while only 17.9% of NF1-associated MPNSTs responded to chemotherapy [111]. These results are likely affected by the low number of sporadic MPNSTs observed.

The role of radiotherapy is controversial, especially considering 10–13% of all MPNSTs are associated with previous irradiation [40,123]. This is particularly concerning for pediatric patients because radiation-induced cancers can take years to arise. Patients with NF1 have also been shown to have a heightened radiation sensitivity compared with other patients, further increasing the risk [124]. The current literature suggests radiation therapy should either only be used postoperatively for positive margin resections and as an adjuvant for large (>5 cm) high-grade tumors or never used at all in NF1-associated tumors because it has not been shown to improve OS [125,126,127,128].

### 3.1. Targeted Therapies for MPNST

As for the treatment of MPNST with targeted therapeutics and/or immunotherapy, no successful treatments currently exist [129,130,131]. However, multiple promising avenues for treatment are currently under evaluation in clinical trials (Table 2). As seen in other sarcomas, both targeted therapies and immunotherapies can use biomarkers to predict whether the drug will be effective prior to treatment. These biomarkers are normally based on either mutations present in the cell or on pathways on which the tumor relies for growth and survival. MPNSTs predominately rely on loss of functional NF1, regardless of etiology, as well as mutations of *TP53*, *CDKN2A*, and PRC2 (in the *SUZ12* and *EED* genes), for their growth and survival. Mutations in *NF1* result in the hyperactivation of Ras signaling, specifically the RAS/RAF/MEK/ERK pathway and the PI3K/AKT/mTOR pathway, making them historically favored targets for developmental therapeutic approaches [132,133]. Several of the clinical trials combine the inhibition of those pathways with additional targets.

Due to the role of RAS in the pathogenesis of NF1, many early efforts to develop targeted therapeutics focused on Ras inhibitors but found little success. RAS was discovered in the early 1980s as the first human oncogene. It was later found to have three major isoforms: H-RAS, K-RAS, and N-RAS. Decades of research have gone into developing drugs selectively targeting RAS function both directly and indirectly [134,135]. For example, efforts to directly target RAS through small molecule inhibitors met with little success as binding pockets where an inhibitor could act could not be found, while GDP and GTP had such high affinities for RAS they could not be outcompeted [136]. Researchers then switched to indirectly inhibiting RAS activity by preventing the protein from localizing in the membrane. For example, farnesyl transferase inhibitors (FTIs) block RAS farnesylation, which is crucial for membrane localization [137]. It was later found that, in the presence of FTIs, K-RAS and N-RAS undergo geranylgeranylation as an alternative route to membrane localization and are resistant to FTI intervention [138]. Numerous other approaches were tried to inhibit RAS activity; however, they repeatedly ran into a variety of roadblocks leading to RAS becoming considered an “undruggable” target. This resulted in a pivot towards targeting proteins downstream of RAS, although many researchers have continued attempting to target RAS itself [139].

In 2024, the pan-RAS inhibitor RMC-7977 was discovered and has been shown to have efficacy in NF1-associated tumors, including MPNSTs in vivo [140,141]. Research has also continued into indirect methods of inhibition such as the use of FTIs in combination with the HMG CoA reductase inhibitor lovastatin to suppress resistance due to geranylgeranylation and shows promise in vitro and in vivo [142]. Though previously considered undruggable, creative solutions and a deeper understanding of RAS-driven cancers have led to potential breakthroughs in targeting RAS itself.

**Figure 1:**
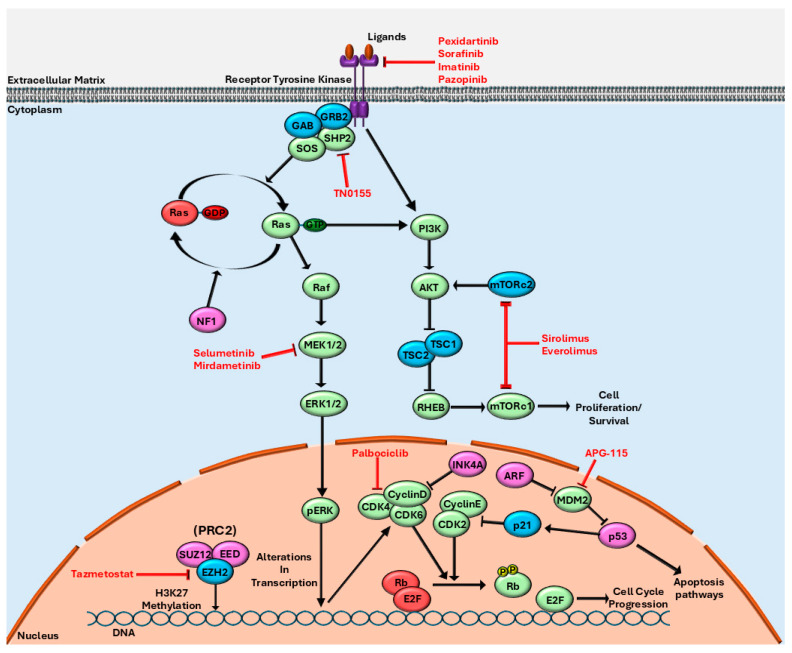
Targetable pathways in malignant peripheral nerve sheath tumors (MPNSTs). Due to common mutations in *NF1*, *CDKN2A* (INK4A, ARF), *TP53* (p53), *SUZ12*, and *EED*, several interrelated pathways have altered activity in MPNSTs. The RAS-related RAF/MEK/ERK and PI3K/AKT/mTOR pathways as well as the CDK4/6 pathway. Epigenetic regulation through PRC2 is also dysregulated. Commonly mutated proteins are depicted in pink, with consequently activated pathways shown in green, and decrease in inactive states of proteins shown in red. Potential therapeutic inhibitors are identified in red font.

MEK inhibitors have been approved for use in plexiform neurofibromas, the benign precursor to NF-1-associated MPNST. MEK is downstream of RAS and its inhibition can block that branch of unregulated RAS signaling. Preclinical studies have shown good efficacy in vitro and in vivo for MPNST cell lines [143,144]. Multiple case studies have shown the success of treating MPNST with *NF1* mutations using MEK inhibitors [145,146]. Tumor cells have been shown to grow resistant to single-agent MEK inhibition, resulting in either a compensatory upregulation of RAS/RAF/MEK/ERK signaling or activity of other pathways such as the mTOR pathway or HGF/MET pathway [147]. A recently completed phase II clinical trial (NCT03433183) tested the MEK inhibitor selumetinib in combination with the mTOR inhibitor sirolimus (Figure 1) and reportedly reached stable diseases in 9 of 21 patients [128].

The use of mTOR inhibitors for MPNST has so far failed to translate successfully into a clinical setting despite preclinical data showing mTOR activation occurring when NF1 is inactivated. A phase II clinical trial of everolimus (Figure 1) and bevacizumab had an ORR of zero and only managed to reach a stable disease in 12% of patients [148]. A phase I/II study showed that ganetespib and sirolimus did not show any response according to the standards of the study even with strong rationale for the combination in a preclinical setting [149]. Currently, an ongoing phase I/II clinical trial (NCT02584647) for pexidartinib, a CSF1R and c-kit inhibitor, plus sirolimus (Figure 1) is looking at outcomes associated with longer treatment compared with the phase I trial. The phase I trial showed a prolonged median PFS compared with other mTOR-based clinical trials, being greater than 18 weeks in 50% of patients, with an OS of 145.1 weeks [150].

Similar to targeting CSF-1R, attempts to target other growth receptors such as VEGFR and PDGFR have generally been unsuccessful. In a trial including 12 patients with MPNST, the VEGFR, RAF, and PDGFR inhibitor sorafenib (Figure 1) achieved a PFS of 1.7 months, an OS of 4.9 months, and reached a stable disease state in 25% of patients [151]. The use of the c-KIT, PDGFR, and VEGFR inhibitor imatinib (Figure 1) allowed stable disease to be reached in one of seven patients, with a PFS of 1.92 months [152]. Another phase II study, using the VEGFR, PDGFR, and c-KIT inhibitor pazopanib (Figure 1), showed slightly more success, achieving a partial response in one patient, with a PFS of 5.4 months and an OS of 10.6 months [153]. A retrospective analysis of pazopanib treatment in patients showed MPNST had a PFS of 6.5 months and an OS of 8.9 months [154]. Across all of these trials, only two patients showed a response to treatment, indicating these agents are unlikely to be beneficial as single-agent therapies. Pazopanib has already been FDA approved for advanced STS in patients previously treated with chemotherapy. Clinical trials studying pazopanib in combination with chemotherapeutics and other targeted therapies for sarcoma are ongoing (NCT06263231, NCT05679921, NCT02180867).

Other work has been conducted looking at the inhibition of the tyrosine phosphatase SHP2, a signaling molecule upstream of RAS. Preclinical studies show improved efficacy in combination with MEK inhibitors compared with MEK inhibitors alone and showed efficacy in an in vitro model of MEK inhibitor-resistant MPNST [155]. Further preclinical research has shown that the anti-tumor efficacy of the SHP2 inhibitor TN0155 (Figure 1) in MPNSTs can be improved when used in combination with CDK4/6 inhibitors [156].

CDK4/6 inhibitors are also a promising target for MPNST therapy. Inactivation of the *CDKN2A/B* gene results in a loss of p16-INK4A and p15-INK4B, which are responsible for the inhibition of the cell cycle through the retinoblastoma (RB1) pathway [157]. RB1 inhibits the G1-S phase transition by sequestering E2F transcription factors, and p16-INK4A and p15-INK4B block the phosphorylation of RB1 by inhibiting the activity of cyclin D-CDK4/6 complexes [157,158,159]. Without functioning p16-INK4A and p15-INK4B, cyclin D-CDK4/6 complexes can stimulate cell cycle progression in a dysregulated manner. At this time no MPNST specific clinical trial has been conducted; however, one trial (EudraCT number: 2016-004039-19) for sarcoma using palbociclib (Figure 1) included a single patient with MPNST and reached a PFS of only 2 months, though promising results were seen in other advanced sarcomas [160].

**Figure 2:**
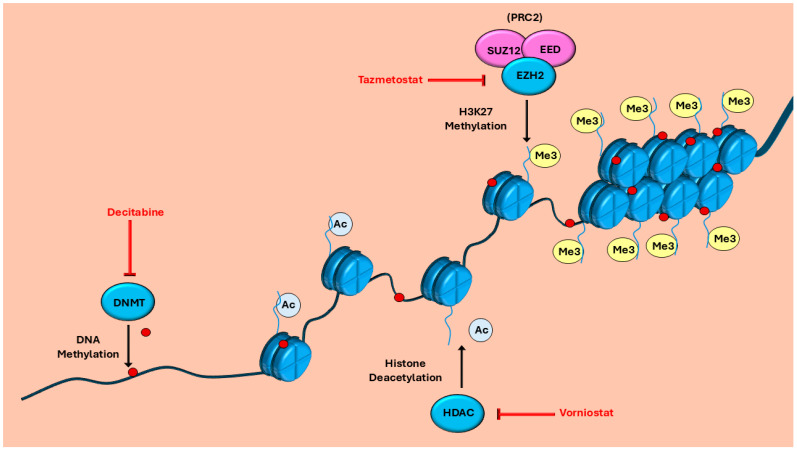
Drugs targeting PRC2 mutations in malignant peripheral nerve sheath tumors (MPNSTs). Mutations in SUZ12 and EED result in the loss of H3K27 trimethylation (yellow), a transcription silencing modification. This results in an increase in histone acetylation (light blue) creating euchromatic DNA or an increase in DNA methylation (red) to keep these regions as heterochromatic DNA. Inhibitors are identified in red font.

Ongoing clinical trials are also attempting to target the loss of PRC2. One such clinical trial (NCT04917042) is using tazemetostat (Figure 1 and Figure 2) to inhibit EZH2 in MPNSTs following its success in other sarcomas. Another trial (NCT04872543) targeting PRC2 uses the drug ASTX727. ASTX727 contains the DNA methyltransferase (DNMT) inhibitor decitabine, which can downregulate oncogene expression, plus the cytidine deaminase inhibitor cedazuridine, which blocks decitabine metabolism. Decitabine (Figure 2) has been shown to increase anti-tumor effects in combination with EZH2 inhibitors and in tumors that lack functioning PRC2 [161]. It has been shown that, due to PRC2 inactivation in MPNST, DNMT1 inhibition has amplified toxicity [162]. This toxicity likely stems from preventing DNMT1 silencing sections of DNA that are regulated by both H3K27 methylation and DNMT1. An upcoming phase 0 clinical trial (NCT06693284) will evaluate the use of mirdametinib (Figure 1) in combination with vorinostat (Figure 2), a histone deacetylase inhibitor, to evaluate efficacy prior to surgery with neoadjuvant radiation therapy. Vornistat prevents H3K27 deacetylation resulting in increased H3K27 acetylation, which is already increased in the absence of PRC2 and has recently been shown to decrease EZH2 activity and expression [163]. Vornistat may be able to increase H3K27 acetylation, leading to the activation of transcription of pro-apoptotic and tumor suppressor genes [164]. Preclinical data also support the use of bromodomain inhibitors targeting bromodomain protein BRD4 in cancers lacking PRC2 function, as well as for its involvement in MEK inhibitor resistance [165,166,167]. BRD4 is a member of the BET family of proteins and is an epigenetic transcription regulator that binds to acetylated histones, helping to recruit transcription machinery, as well as binding lysine-acetylated EED to help facilitate H3K27me3 [168]. Increased reliance on BRD4 by cancer cells to facilitate transcription at H3K27ac sites may create an increased vulnerability to BRD4 inhibitors.

Another upcoming clinical trial in patients with NF1 (NCT06735820) will target different MPNST driver mutations, specifically *TP53* mutations. MPNSTs commonly have alterations in *TP53*; however, they are often a reduction in expression through the loss of heterozygosity instead of the loss of function mutations [8,169,170]. The clinical trial uses selumetinib in combination with APG-115 (Figure 1), an MDM2 inhibitor. MDM2 is an E3 ligase responsible for targeting p53 for degradation; inhibiting its action increases p53 activity and its related cell death pathways [171]. An MDM2 inhibitor recently showed promising efficacy in combination with a PD-1 inhibitor for MPNST treatment during an ongoing clinical trial (NCT03611868) of the combination in a variety of advanced solid tumors [130].

### 3.2. Immunotherapy in MPNST

MPNSTs, like many other sarcomas, are immunologically cold tumors, i.e., they have low immune cell infiltration and have an immunosuppressive tumor microenvironment (TME). The immunosuppressive nature of the TME is in part thought to be facilitated through activated RAS pathway signaling. Activated RAS signaling promotes the infiltration of pro-tumoral tumor-associated macrophages and differentiation of anti-inflammatory regulatory T cells through the secretion of immunosuppressive chemokines and cytokines [172]. The immunosuppressive nature of the MPNST TME creates an environment in which ICIs are not generally effective because the TME lacks the immune cells these drugs activate.

Though ICIs are generally ineffective, limited case reports have shown the successful treatment of MPNSTs [173,174,175]. Since MPNST have been shown to express high amounts of ICI targets such as PD-L1, research with these drugs continues [176,177]. The clinical trial NCT04465643 is using the ICIs nivolumab and ipilimumab as a neoadjuvant treatment for MPNST but has yet to publish results. Other clinical trials are approaching immunotherapy with different approaches. For example, NCT02700230 is evaluating the use of MV-NIS vaccine therapy to induce an effective immune response in MPNSTs. MPNSTs are also included in several ongoing immunotherapeutic studies targeting a wider range of tumors through means such as ICIs (NCT02834013), CAR-T cell therapy (NCT04483778, NCT03618381), CD40 agonists (NCT03719430), and viral therapies (NCT04420975).

## 4. Conclusions

Looking at the advancements made in cancer therapy of other sarcomas, it is surprising how little success has been achieved so far in developing therapeutics for MPNSTs. This could be attributed to the rarity of the disease and the difficulty of studying it; yet, other even rarer sarcomas have had successful treatments developed. It is more likely that the complex heterogeneity of MPNSTs is limiting progress in the translation of therapeutics to the clinic. We lack the understanding of why treatments for MPNSTs repeatedly fail to show the expected results in a clinical setting after promising preclinical data. The successes in other sarcomas have shown that the development of therapeutics for unresectable and metastatic disease is built on an understanding of the biology of the tumor, with most successful drugs targeting driver mutations specific to that type of sarcoma. In the case of MPNSTs, it is hard to develop this understanding due to the limited population that can be observed, as well as high levels of heterogeneity. Recently there has been a shift to targeting tumor-driving mutations besides the mutation in NF1 and its resultant activation of the RAS pathways. Other efforts have focused on gaining an understanding of why MPNSTs display resistance to ICIs and Ras/Raf/MEK/ERK pathway blockades, how to overcome resistance through combination therapy, and creating models that better reflect the heterogeneity of the patient population. Going forward it is imperative that the current understanding of the major driver mutations of MPNST such as *NF1*, *CDKN2A/B*, PRC2, and *TP53* is expanded. In particular, we need to understand how these drivers interact to control the state of the tumor to gain a more comprehensive understanding of these pathways and identify therapeutic vulnerabilities that may require multiple targets. The shifting approach in how therapeutics are being developed for MPNSTs, mirroring successes in other sarcomas, provides an optimistic outlook for breakthroughs in the treatment of these tumors.

## Figures and Tables

**Table 1:** Successful targeted therapies in sarcoma and related tumors.

Drug	Mechanism of Action	Disease Treated
Imatinib	Multi-kinase inhibitor	Gastrointestinal Stromal Tumors
Sunitinib	Multi-kinase inhibitor	Gastrointestinal Stromal Tumors
Regorafinib	Multi-kinase inhibitor	Gastrointestinal Stromal Tumors
Ripretinib	Multi-kinase inhibitor	Gastrointestinal Stromal Tumors
Avapritinib	Multi-kinase inhibitor	Gastrointestinal Stromal Tumors with a PDGFRA D842V Mutation
Crizotinib	ALK inhibitor	Inflammatory Myofibroblastic Tumor
*nab*-sirolimus	mTOR inhibitor	Perivascular Epithelioid Tumor
Pomalidomide	Angiogenesis inhibitor	Kaposi Sarcoma
Tazemetostat	EZH2 inhibitor	Epithelioid Sarcoma
Nirogacestat	γ-Secretase Inhibitor	Desmoid Tumors
Selumetinib	MEK inhibitor	Inoperable Plexiform Neurofibromas
Mirdametinib	MEK inhibitor	Inoperable Symptomatic Plexiform Neurofibromas
Pexidartinib	CSF-1R inhibitor	Tenosynovial Giant Cell Tumor

**Table 2:** Upcoming or recent clinical trials targeting MPNSTs.

Intervention	Mechanism of Action	Phase	Target Tumors	Clinicaltrials.gov ID
Pexidartinib and Sirolimus	CSF-1R and mTOR inhibitors	I/II	Unresectable sarcoma and MPNSTs	NCT02584647
Selumetinib and Sirolimus	MEK and mTOR inhibitors	II	Unresectable or metastatic MPNSTs	NCT03433183
Selumetinib and APG-115	MEK and MDM2 inhibitors	0/1/2	Refractory or unresectable MPNST or ANNUBPs	NCT06735820
Mirdametinib and Vorinostat	MEK and histone Deacetylation inhibitors	0	Primary MPNST pre-resection.	NCT06693284
Tazemetostat	EZH2 inhibitor	II	Recurrent or metastatic MPNSTs	NCT04917042
ASTX727	DNA methyl-transferase inhibitor	II	MPNST with a PRC2 mutation	NCT04872543
Nivolumab and Ipilimumab	PD-1 and CTLA-4 inhibitors	I	Pre-malignant and MPNSTs	NCT04465643
MV-NIS	Vaccine therapy	I	Unresectable or recurrent MPNSTs	NCT02700230

## Abbreviations

The following abbreviations are used in this manuscript:

MPNST	malignant peripheral nerve sheath tumor
NF1	neurofibromatosis type 1
STS	soft tissue sarcoma
OS	overall survival
RIS	radiation-induced sarcoma
ORR	objective response rate
PFS	progression free survival
TKI	tyrosine kinase inhibitor
PKI	protein kinase inhibitor
GIST	gastrointestinal stromal tumor
IMT	inflammatory myofibroblastic tumor
TGCT	tenosynovial giant cell tumor
PEComa	perivascular epithelioid cell tumor
*nab*-sirolimus	nanoparticle albumin-bound sirolimus
TSC	tuberous sclerosis complex
KSHV	Kaposi sarcoma-associated herpesvirus
TME	tumor microenvironment
ICI	immune checkpoint inhibitor
ASPS	alveolar soft part sarcoma
ACT	adoptive cell therapy
FTI	farnesyl transferase inhibitor
